# ﻿A new species of the genus *Achalinus* (Squamata, Xenodermatidae) from southwest Hunan Province, China

**DOI:** 10.3897/zookeys.1189.112784

**Published:** 2024-01-17

**Authors:** Hui Li, Le-Qiang Zhu, Bei Xiao, Jie Huang, Shao-Wu Wu, Li-Xun Yang, Zhi-Qiang Zhang, Xiao-Yang Mo

**Affiliations:** 1 Vertebrate Zoology Laboratory, College of Life Science, Hunan Normal University, Changsha 410081, China Hunan Normal University Changsha China; 2 College of Biology Resources and Environmental Sciences, Jishou University, Jishou 416000, China Jishou University Jishou China; 3 Forestry Bureau of Tongdao Dong Autonomous County, Huaihua 418500, China Forestry Bureau of Tongdao Dong Autonomous County Huaihua China; 4 Institute of Wildlife Conservation, Central South University of Forestry and Technology, Changsha 410004, China Central South University of Forestry and Technology Changsha China

**Keywords:** *Achalinusnanshanensis* sp. nov., molecular analyses, morphology, taxonomy

## Abstract

A new species of xenodermid snake, *Achalinusnanshanensis* H. Li, L.-Q. Zhu, Z.-Q. Zhang & X.-Y. Mo, **sp. nov.**, is described based on three specimens collected from Nanshan National Park and Tongdao County of southwest Hunan Province. This new species is genetically distinct amongst its congeners with the mitochondrial COI uncorrected *p*-distance ranging from 4.4% (in *A.yangdatongi*) to 17.7% (in *A.meiguensis*). In addition, this new species can be distinguished from its congeners by a combination of the following morphological characters: (1) dorsal scales with 23 or 25 rows throughout and strongly keeled; (2) tail relatively longer so that TaL/ToL = 0.215–0.248; (3) length of suture between internasals significantly longer than that between prefrontals, LSBI/LSBP = 1.66–1.84; (4) single loreal scale present; (5) SPL 6 in number, with the fourth and fifth contacting eye; (6) IFL 6 in number, with the first three touching the first pair of chin shields; (7) TMP is 2-2-4/2-2(3)-4, with the anterior pair elongated and in contact with the eye; (8) ventrals 2 + 147–158; (9) subcaudals 64–77, unpaired; (10) dorsal body brownish black, with a bright yellow neck collar extending to the head and abdomen in the occipital region. The recognition of the new species increases the number of described *Achalinus* species to 28, of which 21 are found in China.

## ﻿Introduction

Xenodermidae is composed of six known genera (*Xenodermus* Reinhardt, 1836, *Achalinus* Peters, 1869, *Stoliczkia* Jerdon, 1870, *Fimbrios* Smith, 1921, *Parafimbrios* Teynié, David, Lottier, Le, Vidal & Nguyen, 2015, and *Paraxenodermus* Deepak, Lalronunga, Lalhmingliani, Das, Narayanan, Das & Gower, 2021). The genus *Achalinus* is the most diverse genus of the family Xenodermidae (Uetz et al. 2022). It contains 27 recognized species and is widely distributed in eastern and southeastern Asia, where it ranges from northern Vietnam to southwestern China and partly into Japan. In the past five years, 18 species have been described in this genus (Wang et al. 2019; Ziegler et al. 2019; Li et al. 2020, 2021; Luu et al. 2020; Miller et al. 2020; Hou et al. 2021; Huang et al. 2021; Ha et al. 2022; Yang et al. 2022, 2023; Zhang et al. 2023; Ma et al. 2023a, 2023b). However, because of unresolved taxonomic problems and the poorly known distribution patterns of many species, the diversity of this genus remains underestimated.

During recent herpetological surveys in southwest Hunan, China, three *Achalinus* snake specimens were collected in Tongdao County and Nanshan National Park. The specimens exhibit the morphological characteristics of the genus *Achalinus*, which include a small, elongated, cylindrical body; strongly keeled, lanceolate scales with a metallic luster; and the lack of preocular and postocular scales, featuring a single loreal scale and temporals that are in direct contact with the eyes (Peters, 1869; Zhao et al. 1998; Zhao, 2006), but they could not be assigned to any known species. Extensive morphological examinations and further molecular analyses revealed that these specimens represent a separately evolving lineage within the genus *Achalinus* and can be distinguished from recognized congeners. We herein describe this overlooked *Achalinus* population as a new species, based on an iterative taxonomic approach.

## ﻿Materials and methods

### ﻿Sampling

**Figure 1.**
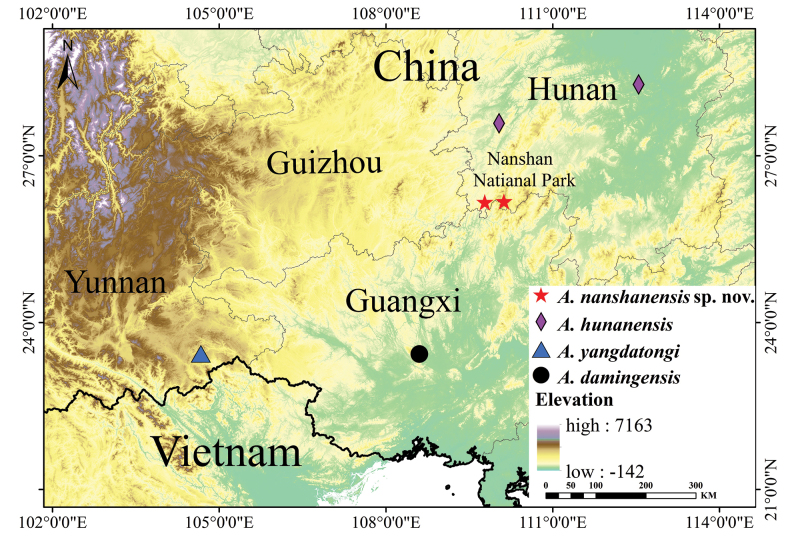
Distribution of some species of the genus *Achalinus*, Red star: the type locality of *A.nanshanensis* sp. nov. (HNNU230902, HNNU230903): Nanshan Nation Park, Shaoyang City, Hunan Province, China; *A.nanshanensis* sp. nov. (HNNU230901): Tongdao County, Huaihua City, Hunan Province, China. Blue triangle: the type locality of *A.yangdatongi* in Xichou County, Yunnan Province; Black circle: the type locality of *A.damingensis* in Shanglin County, Guangxi Province. Purple diamond: the type locality of *A.hunanensis* in Hecheng District and Ningxiang County, Hunan Province.

Three odd-scaled snake specimens were collected in Hunan Province, China: two specimens (HNNU230902, HNNU230903) were collected in Nanshan National Park, and one specimen (HNNU230901) was collected in Tongdao County (Fig. 1). The three specimens were collected in the field, fixed in 75% ethanol, and deposited in the Vertebrate Zoology Laboratory, College of Life Science, Hunan Normal University. For molecular analyses, 33 sequences were used, among which 30 (sequences 4–33 in Table 1) were obtained from GenBank which include 27 sequences of 23 *Achalinus* species. Additionally, sequences of *Fimbriosklossi*, Smith, 1921, *Parafimbrioslao*, Teynié, David, Lottier, Le, Vidal & Nguyen, 2015 and *Xenodermusjavanicus*, Reinhardt, 1836 were used as outgroups. Details are shown in Table 1.

**Table 1.** Localities, voucher information, COI GenBank accession numbers, and references for all samples used in this study.

No.	Species	Voucher	Locality	GenBank	References
1	*A.nanshanensis* sp. nov.	HNNU230901	Tongao, Huaihua, Hunan, China	OR523368	This study
2	*A.nanshanensis* sp. nov.	HNNU230902	Nanshan National Park, Hunan, China	OR523369	This study
3	*A.nanshanensis* sp. nov.	HNNU230903	Nanshan National Park, Hunan, China	OR523370	This study
4	*A.ater*	SYSr00852	Huaping Nature Reserve, Guangxi, China	MN380334	Wang et al. 2019
5	*A.dabieshanensis*	AHU2018EE0710	Fuziling Provincial Reserve, Anhui, China	MW316598	Zhang et al. 2023
6	*A.damingensis*	ANU20220009	Shanglin, Nanning, Guangxi, China	OP644487	Ynang et al. 2023
7	*A.dehuaensis*	YBU13013	Dehua, Fujian, China	MZ442642	Li et al. 2021
8	*A.emilyae*	IEBR4465	HoanhBo, Quang Ninh, Vietnam	MK330857	Ziegler et al. 2019
9	*A.formosanus*	RN2002	Taiwan, China	KU529452	Unpublished
10	*A.huangjietangi*	HSR18030	Huangshan, Anhui, China	MT380191	Huang et al. 2021
11	*A.hunanensis*	CIB119039	Huaihua, Hunan, China	OQ848425	Ma et al. 2023
12	*A.hunanensis*	CIB119040	Ningxiang, Hunan, China	OQ848426	Ma et al. 2023
13	*A.juliani*	IEBRA.2018.8	HaLang, Cao Bang, Vietnam	MK330854	Ziegler et al. 2019
14	*A.meiguensis*	GP835	Mianyang, Sichuan, China	MZ442641	Li et al. 2021
15	*A.niger*	RN0667	Taiwan, China	KU529433	Unpublished
16	*A.ningshanensis*	ANU20220006	Ningshan, Shaanxi, China	ON548422	Yang et al. 2022
17	*A.ningshanensis*	ANU20220007	Ningshan, Shaanxi, China	ON548423	Yang et al. 2022
18	*A.panzhihuaensis*	KIZ040189	Yanbian, Sichuan, China	MW664862	Hou et al. 2021
19	*A.pingbianensis*	YBU18273	Honghe, Yunnan, China	MT365521	Li et al. 2020
20	*A.quangi*	sp4	northern Vietnam	OQ197471	Anh et al. 2023
21	*A.rufescens*	SYSr001866	Hongkong, China	MN380339	Wang et al. 2019
22	*A.spinalis*	SYSr001327	Badagong Mountains, Hunan, China	MN380340	Wang et al. 2019
23	*A.timi*	IEBRA.2018.10	ThuanChau, Son La, Vietnam	MK330856	Ziegler et al. 2019
24	*A.tranganensis*	VNUFR.2018.21	NinhBinh, Vietnam	MW023086	Luu et al. 2020
25	*A.vanhoensis*	VNUFR.2019.13	VanHo, Son La, Vietnam	ON677935	Ha et al. 2022
26	*A.yangdatongi*	KIZ034327	Wenshan Nature Reserve, Yunnan, China	MW664865	Hou et al. 2021
27	*A.yangdatongi*	YPX51447	Xichou county, Yunnan, China	MW664864	Xu et al, 2023
28	*A.yangdatongi*	YPX51446	Xichou county, Yunnan, China	MW664863	Xu et al, 2023
29	*A.yunkaiensis*	SYSr001443	Dawuling Forestry Station, Guangdong, China	MN380329	Wang et al. 2019
30	*A.zugorum*	IEBR4698	Bac Me, Ha Giang, Vietnam	MT502775	Miller et al. 2020
31	*Fimbriosklossi*	IEBR3275	Quang Ngai, Vietnam	KP410744	Teynié et al. 2015
32	*Parafimbrioslao*	MNHN2013.1002	Louangphabang, Laos	KP410746	Teynié et al. 2015
33	*Xenodermusjavanicus*	–	Sumatera Barat, Indonesia	KP410747	Teynié et al. 2015

### ﻿Morphological examination

Morphological descriptions follow Zhao (2006). Abbreviations in this study are as follows: ,snout–vent length (**SVL**, length from tip of snout to anterior margin of the cloaca); ,tail length (**TaL**, length from posterior margin of cloaca to tip of tail); ,total length (**ToL**, length from snout tip to tail end; ,head length (**HL**, length from the tip of snout to the posterior margin of mandible; ,head width (**HW**, width from the widest part of the head in dorsal view); ,eye diameter (**ED**, diameter from the most anterior corner of the eye to the most posterior corner); ,loreal height (**LorH**, height from the highest part to the lowest part of the loreal in lateral view); ,loreal length (**LorL**, length from the most anterior loreal to the most posterior loreal in lateral view); ,length of the suture between internasals (**LSBI**); ,length of the suture between prefrontals (**LSBP**). Three characters were measured with a ruler to the nearest 1 mm: **SVL**, **TaL**, and **ToL**; other measurements were measured used digital calipers to the nearest 0.1 mm.

The scale features and their abbreviations are as follows: ,loreals (**Lor**); ,supralabials (**SPL**); ,infralabials (**IFL**); ,number of chin shield pairs (**Chins**); infralabials touching the first pair of chin shields (**IFL-1^st^ Chin**); ,postoculars (**PtO**); ,temporals (**TMP**); ,supraoculars (**SPO**); ,temporals (**TEM**), ,number of anterior temporals that touch the eye (**aTEM-Eye**) (head bilateral scale counts are given as left/right), ,pre-ventral scales (**PrV**), ,ventral scales (**VEN**), ,subcaudal (**SC**), ,entire or divided state of the anal scales (**Anal**), ,dorsal scale rows (**DSR**) (counted at one-head-length behind the head, at midbody, and at one-head-length before the anal); ,the number of maxillary teeth (**MT**). We also make comparisons with other species of the genus *Achalinus* based on available literature (Peters 1869; Boulenger 1888, 1908; Van 1912; Pope 1935; Bourret 1937; Hu and Zhao 1966; Hu et al. 1975; Zong and Ma 1983; Ota and Toyama 1989; Zhao et al. 1998; Zhao 2006; Wang et al. 2019; Ziegler et al. 2019; Li et al. 2020; Luu et al. 2020; Miller et al. 2020; Hou et al. 2021; Huang et al. 2021; Ha et al. 2022; Yang et al. 2022, 2023; Ma et al. 2023a, 2023b; Xu et al. 2023; Zhang et al. 2023). The sex was determined by the presence/absence of everted hemipenes.

### ﻿Phylogenetic analyses

Genomic DNA was extracted from preserved liver tissue using the TIANamp Genomic DNA Kit. The fragment of the mitochondrial DNA gene encoding cytochrome c oxidase subunit I (COI) was amplified using the primer pairs Chfm4 and Chrm4 (Che et al. 2012). The polymerase chain reaction (PCR) was performed in 20 μL of reactant with the following cycling conditions: 95 °C for 4 min, 35 cycles of denaturing at 94 °C for 40 s, annealing at 53 °C for 40 s, and extending at 72 °C for 1 min, and a final extending step of 72 °C for 10 min. The PCR products were sequenced at Shanghai Map Biotech Co., Ltd.

The COI sequences (629 bp) were assembled using SeqMan in the DNASTAR software package (Burland 2000), and compared and aligned using MEGA 7 software (Kumar et al. 2018). The uncorrected pairwise distances (*p*-distance) were calculated in MEGA 7. MrBayes 3.2.4 (Ronquist et al. 2012) was used to conduct the Bayesian inference analysis under the best-fitting model GTR + I + G4, which was selected by ModelFinder identified via AICc (Darriba et al. 2012). A maximum-likelihood analysis (Nguyen et al. 2015) was executed using IQ-TREE 2 under the best-fit model TIM3 + F + I + G4 selected by Modelfinder according to AICc.

## ﻿Results

### ﻿Molecular analyses

**Figure 2.**
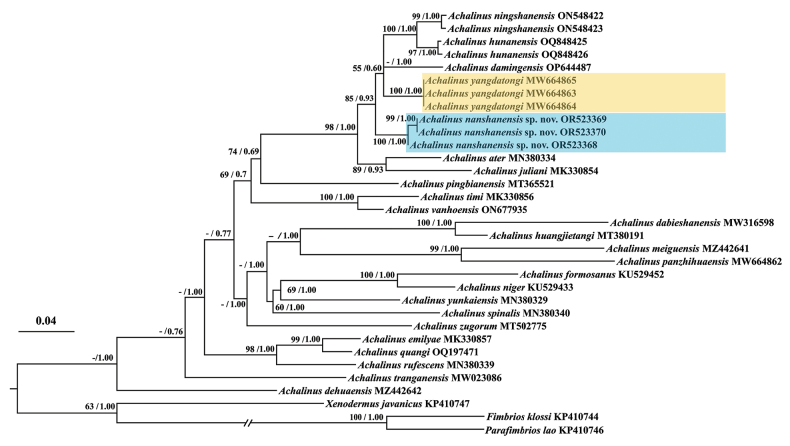
Phylogenetic tree of the genus *Achalinus* inferred from CO1 gene fragments (629 bp) by maximum-likelihood analysis. The numbers above the branches represent the supporting values: SH-like approximate likelihood ratio test and Bayesian posterior probabilities (the ones lower than 50 are displayed as “-”). *Achalinusnanshanensis* sp. nov. is highlighted in blue and *A.yangdatongi* in yellow.

The maximum-likelihood (ML) and Bayesian-inference (BI) analyses resulted in essentially identical topologies, which are integrated in Fig. 2 with the distances given in Table 2. The new species is nested within the genus *Achalinus*, and its affinity to *A.yangdatongi*, *A.damingensis*, *A.ningshanensis*, and *A.hunanensis* considerably strong supported (BI, PP = 0.85; ML, BS = 93%). In addition, the *p*-distance among all species within the genus ranges from 4.4–17.7% (Table 2), the minimum genetic distance between the new species and its congers is greater than the lowest one (3.2–3.4% between *A.ningshanensis* and *A.hunanensis*). Given that the *Achalinus* populations from Tongdao County and Nanshan National Park possess significant morphological differences from all known congeners, we describe it as a new species below.

**Table 2.** Uncorrected *p*-distances (%) among *Achalinus* species inferred from mitochndrial COI gene.

	1–3	4	5	6	7	8	9	10	11–12	13	14	15	16–17	18	19	20	21	22	23	24	25	26–28	29
1–3 *A.nanshanensis* sp. nov.	0–0.5																						
4 *A.ater*	6.7–6.9																						
5 *A.dabieshanensis*	16.0	14.7																					
6 *A.damingensis*	5.3–5.8	8.2	15.8																				
7 *A.dehuaensis*	14.3–14.6	16.5	18.4	16.0																			
8 *A.emilyae*	12.8–13.0	11.7	17.7	13.0	15.5																		
9 *A.formosanus*	14.4–14.9	14.1	19.0	14.9	15.9	13.9																	
10 *A.huangjietangi*	16.6	15.0	8.9	16.3	16.5	14.5	15.6																
11–12 *A.hunanensis*	4.7–5.6	7.1–7.3	16.9–17.1	6.1–6.3	14.9–15.3	13.0–13.2	13.7–14.0	16.8	0.5														
13 *A.juliani*	7.7–7.9	7.1	15.8	8.5	14.9	12.3	12.5	14.6	8.7–8.8														
14 *A.meiguensis*	17.7	15.4	17.7	16.8	18.1	15.4	15.6	15.2	16.4	16.8													
15 *A.niger*	12.8–13.3	13.5	15.8	14.3	15.9	12.2	9.1	13.9	13.2–13.3	12.3	13.9												
16–17 *A.ningshanensis*	5.1–5.6	7.6–7.7	17.1–17.2	7.2–7.8	16.3–16.5	13.5–14.1	14.8–15.1	17.2	**3.2–3.4**	9.1–9.6	17.0	14.6	0.7										
18 *A.panzhihuaensis*	15.1–15.5	16.2	16.6	15.5	15.3	16.6	16.0	15.2	16.2	15.5	11.6	14.4	17.1–17.4										
19 *A.pingbianensis*	11.6–11.8	11.8	15.3	11.3	14.9	12.9	14.6	13.0	11.1	12.1	16.8	11.8	11.7–12.4	14.9									
20 *A.quangi*	12.8–13.1	11.7	18.1	13.1	15.4	**3.5**	13.9	15.0	13.2	12.7	15.2	13.4	12.8–13.4	16.9	13.9								
21 *A.rufescens*	11.9–12.0	12.7	16.9	13.8	14.3	8.0	14.1	14.3	12.1	12.3	17.3	12.3	12.3–12.7	16.0	12.9	7.9							
22 *A.spinalis*	13.9–14.1	15.2	16.6	15.1	14.3	13.9	13.9	13.4	13.9	13.9	16.0	15.6	15.1–15.6	15.8	13.3	13.9	13.0						
23 *A.timi*	13.6–13.8	13.3	16.4	13.5	16.0	13.1	13.8	14.8	12.0	14.1	15.8	13.6	13.6	15.5	12.3	13.6	13.9	14.3					
24 *A.tranganensis*	13.0–13.1	12.7	15.3	13.9	13.9	11.5	16.8	13.4	14.0	13.3	16.4	15.2	14.3–15.2	16.4	13.3	12.2	11.5	14.6	13.8				
25 *A.vanhoensis*	12.4–12.8	13.1	15.5	12.6	16.0	12.2	14.0	14.6	11.5	13.6	15.6	12.1	12.1–12.4	15.5	10.8	12.4	13.8	12.9	5.2	13.3			
26–28 *A.yangdatongi*	**4.4**	6.2	16.6	5.6	14.0	12.8	14.4	14.6	5.1	7.3	17.1	5.9	5.8–5.9	15.5	11.3	12.6	11.5	14.2	13.1	12.8	11.3		
29 *A.yunkaiensis*	12.5–12.8	12.8	14.9	12.5	14.7	13.1	12.3	12.5	12.0	12.5	15.8	13.7	13.0–13.7	15.7	11.6	13.6	13.3	12.0	14.1	13.5	13.6	12.0	
30 *A.zugorum*	12.8–13.0	13.1	15.3	12.8	14.3	12.3	13.1	14.3	11.8	13.1	15.0	12.8	12.8	15.3	11.0	12.7	13.5	13.3	13.6	11.9	11.9	12.2	10.9

### ﻿Taxonomic account

#### ﻿ Achalinus nanshanensis

Taxon classificationAnimaliaSquamataXenodermatidae

H. Li, L.-Q. Zhu, Z.-Q. Zhang & X.-Y. Mo sp. nov.

616C4808-7946-5F1A-9DE8-53320425A974

https://zoobank.org/353AD101-0B8D-4C85-88FE-0E0C63120051

Fig. 3 , Table 3

##### Type materials.

***Holotype***: China • adult ♂; Hunan Province, Shaoyang City, Chengbu County, Nanshan National Park; 26°11′46.34″N, 110°07′56.38″E, alt. 1665 m; 1 Sept. 2023; Hui Li & Leqiang Zhu leg.; HNNU230903. ***Paratypes***: China • 1 adult ♂; Hunan Province, Huaihua City, Tongdao County; 25°54′42.37″N, 109°44′31.39″E; alt. 300 m; 14 Oct. 2022; Shaowu Wu & Lixun Yang leg.; HNNU230901 • 1 adult ♂; same locality and date as holotype; HNNU230902.

**Figure 3.**
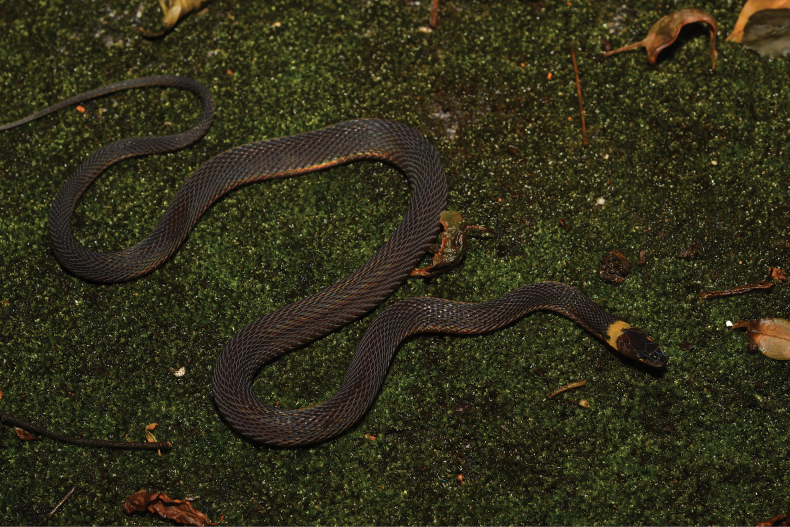
General view of *Achalinusnanshanensis* sp. nov. (HNNU230902) in life. Photo by Le-Qiang Zhu.

##### Etymology.

The new species is named for on its type locality. We suggest the Chinese common name 南山脊蛇 (pin yin: Nán Shān Jǐ Shé) and the English common name Nanshan odd-scaled snake.

**Table 3.** Main morphological characters of *Achalinusnanshanensis* sp. nov.

	Voucher		
	HNNU230903	HNNU230901	HNNU230902
	Holotype	Paratype	Paratype
**Sex**	Adult male	Adult male	Adult male
**SVL**	362	302	300
**TaL**	99	99	99
**TL**	461	401	399
**TaL/TL**	0.215	0.247	0.248
**HL**	10.95	10.07	10.18
**HW**	7.25	5.96	6.37
**ED**	1.11	1.10	1.09
**SPL**	6/6	6/6	6/6
**SPL-Eye**	4^th^–5^th^	4^th^–5^th^	4^th^–5^th^
**IFL**	6	6	6
**Chin**	2	2	2
**IFL-1^st^Chin**	1^st^–3^rd^	1^st^–3^rd^	1^st^–3^rd^
**SPO**	1	1	1
**LorH**	0.83	0.77	0.72
**LorL**	1.86	1.45	1.46
**LorH / LorL**	0.47	0.53	0.49
**LSBI**	1.78	1.52	1.49
**LSBP**	1.07	0.86	0.81
**LSBI / LSBP**	1.66	1.76	1.84
**TEM**	2+2+4	2+2+4	2+2(rarely 3)+4
**aTEM-Eye**	2	2	2
**DSR**	23-23-23	23-23-23	25-25-25
**VEN**	155	158	147
**SC**	64	77	72
**Anal**	1	1	1

##### Diagnosis.

The new species can be distinguished from other members of *Achalinus* by the following characteristics: (1) dorsal scales with 23 or 25 rows throughout and strongly keeled; (2) tail relatively longer so that TaL/ToL = 0.215–0.248; (3) length of suture between internasals significantly longer than that between prefrontals, LSBI/LSBP = 1.66–1.84; (4) single loreal scale present; (5) SPL 6 in number, with the fourth and fifth contacting eye; (6) IFL 6 in number, with the first three touching the first pair of chin shields; (7) TMP is 2-2-4/2-2(3)-4, with the anterior pair elongated and in contact with the eye; (8) ventrals 2 + 147–158; (9) subcaudals 64–77, unpaired; (10) dorsal body brownish black with a bright-yellow neck collar extending to the head and abdomen in the occipital region.

##### Description of holotype.

Adult male with a total length of 461 mm (SVL 362 mm and TaL 99 mm), tail relatively long, TaL/ToL 0.215, body slender and cylindrical. Head distinct from neck, rostral small, triangular, only upper tip visible from above. Head length 10.95 mm, head width 7.25 mm. Length of suture between internasals much longer than that between prefrontals (LSBI 1.78 mm, LSBP 1.07 mm, LSBI/LSBP 1.66). Frontal pentagonal pointed backwards, much shorter than parietals; each parietal bordered with an elongated nuchal, with no preoculars and postoculars. Nostril at anterior part of nasal scale, posterior margin of nostril with a distinct nostril cleft. A single loreal scale present, extending from nasal scale to eye, distinctly wider than high. Eyes small, ED 1.11 mm. Two aTMP and four pTMP present; aTMPs elongated, upper one much smaller than the lower one; upper one in contact with eye, lower one also in contact with parietal scale. SPL 6 in number, the fourth and fifth in contact with the eye, the sixth longest. Two pairs of shields present, the first three in contact with first chin shield. One mental scale present, the first IFL in contact with each other after the mental scale, followed by another 5 IFL in contact with each other. Dorsal scales 23-23-23, strongly keeled; dorsum with no longitudinal vertebral stripe. VEN 155 in number. SC 64 in number, uniserial, anal entire.

##### Coloration of holotype in life.

Scales possess a subtle iridescent quality. The dorsum’s distinguishing characteristic is its reflective, brownish-black appearance, with a notable bright-yellow patch that extends to the head and abdomen in the occipital region. The first pair of chin shields displays black coloration at the front, while the second pair is entirely white. The eyes are uniformly black. The ventral side is prevalently grayish white, with the edges of the ventral scales gradually transitioning from grayish white to black. The ventral coloration of the tail mirrors that of the dorsum, featuring a brownish-black hue.

##### Coloration in preservative.

**Figure 4.**
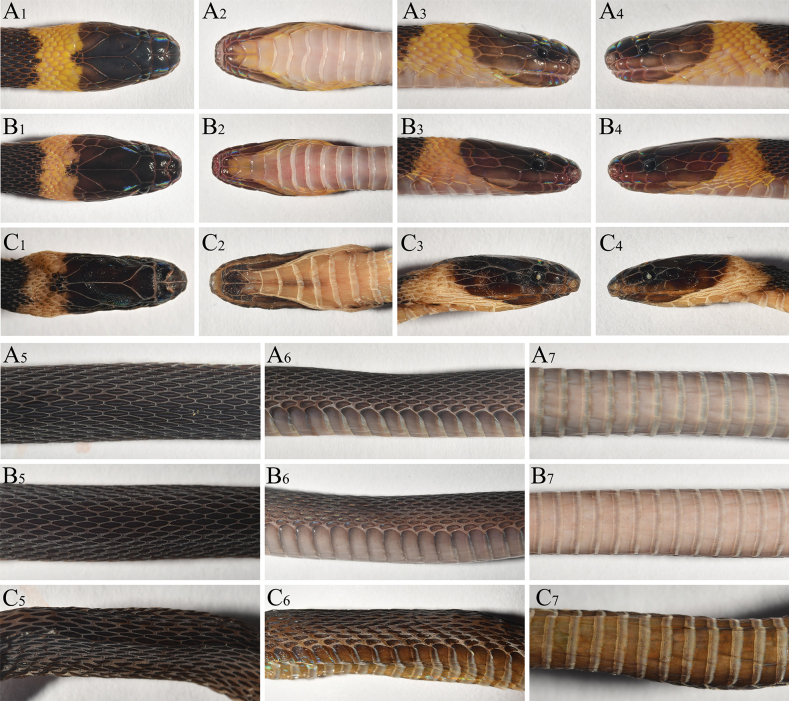
The view of *Achalinusnanshanensis* sp. nov. **A** HNNU230903 (holotype) **B** HNNU230902 (paratype) **C** HNNU230901 (paratype), remaining photos by Le-Qiang Zhu.

**Figure 5.**
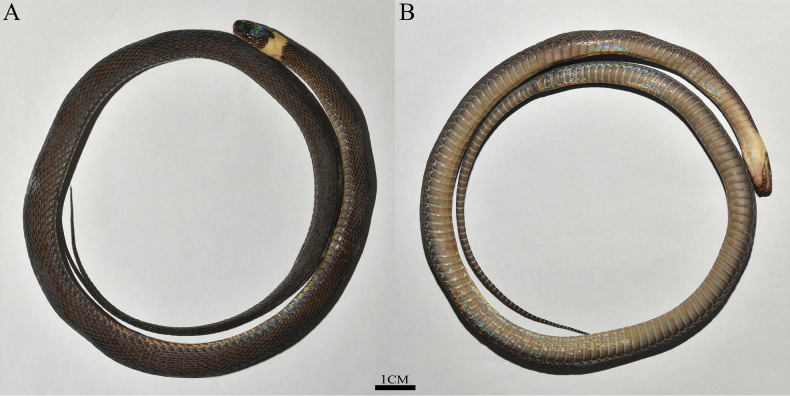
Holotype of *Achalinusnanshanensis* sp. nov. (HNNU230903) in preservative **A** dorsal view **B** ventral view. Photos by Hui Li.

(Figs 4, 5) All scales retain a subtle iridescence. The coloration darkens as it transitions from the dorsum to the venter, with the dorsal surface of the body primarily appearing brownish black. Notably, the collar of the neck is a paler grayish white.

##### Distribution and habits.

**Figure 6.**
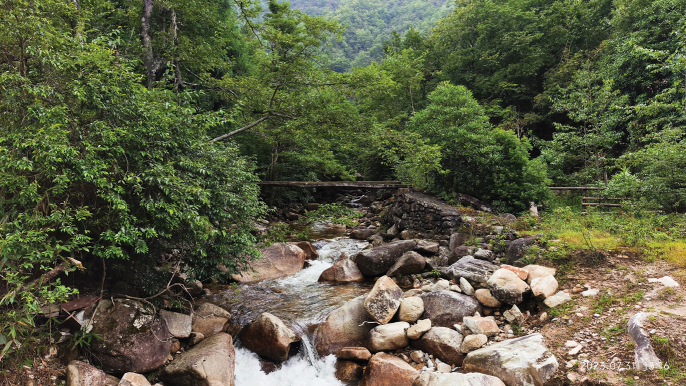
Habitat of *Achalinusnanshanensis* sp. nov. (HNNU230902, HNNU230903), Nanshan National Park, Chengbu County, Shaoyang City, Hunan Province, China. Photo by Le-Qiang Zhu.

(Fig. 6) *Achalinusnanshanensis* sp. nov. is currently known from Hunan Province, China, and specifically from Nashan National Park, Shaoyang City, and Tongdao County in Huaihua City. It has a known elevational range of 300–1665 m above sea level. All three specimens were found during the night, with the holotype and one paratype found near a mountain stream where the air temperature was 18 °C and the relative humidity was 80%. These individuals were close to shrubs in a subtropical broadleaved evergreen forest. They were found making their way from leaf litter to the stream. The other specimen was found in a bamboo forest near a steam.

##### Comparison.

A summary of morphological characteristics is listed in Table 4. Phylogenetically, *A.nanshanensis* sp. nov. is closest to *A.yangdatongi* Hou, Wang, Guo, Chen, Yuan & Che, 2021 and *A.damingensis* Xu, Yang, Wu, Gong, Huang & Huang, 2023. However, *A.nanshanensis* sp. nov. differs from *A.yangdatongi* in having fewer ventral scales (147–158 vs 155–177), maxillary teeth (18 vs 24–26) and more temporals (2+2/3+4 vs 2+2/3+2/3), shorter loreal (0.47–0.53 vs 0.57) (Table 5). The new species differs from *A.damingensis* in having more temporals (2-2/3-4 vs 2-2-3) and fewer ventral scales (147–158 vs 165).

**Table 4.** A morphological comparison of male species of the genus *Achalinus*. Morphological data of examined congeners were taken from the literature. “–” & “?” = unavailable, “*” = female species (sevral species of genus *Achalinus* recorded only female samples).

Species	TaL/ToL	MT	LorH/LorL	LSBI vs. LSBP	DSR	Lor	PtO	SPL	SPL-Eye	IFL	IFL-1st Chin	TEM	aTEM-Eye	VEN	SC
*nanshanensis* sp. nov.	0.215–0.248	18	0.47–0.53	>1	(23–25)-(23–25)-(23–25)	1	0	6	4–5	6	1–3	2+2+4/2+2(3)+4	2	147–158	64–77
*A.ater*	0.190 ~ 0.220	–	0.40	> 1	(21–23)-(21–25)-(21–25)	1	0	6	4–5	5–6	1–3	2+2+3	2	146–164	50–66
*A.dabieshanensis*	0.177 ~ 0.223	–	0.73 ~ 0.81	< 1	23-23-23	1	0	6	4–5	5	1–3	2+2+4	2	141–151	46–55
*damingensis*	427	–	–	>1	23-23-23	1	0	6	4–5	6	1–3	2+2+3	2	165	74
*A.dehuaensis*	0.262 ~ 0.286	31–33	–	> 1	23-23-23	1	0	6	4–5	5	1–3	2+2(3)+3(4)	1–2	142–143	74–81
A.emilyae*	0.183 ~ 0.203	27–28	–	> 1	23-23-23	1	0	6	4–5	5	1–3	2+2+3	1	157–161	56–63
*A.formosanus*	0.159	17	–	= 1	29(27)-27-25	0	0	6	4–5	6–7	–	2+2	1	158–184	61–83
A.hainanus*	0.258 ~ 0.266	–	–	= 1	23-23-23	1	0	6	4–5	5	1–3	1+2+3(4)	1	165–168	67–69
*A.huangjietangi*	0.211 ~ 0.232	–	0.70 ~ 0.74	< 1	23-23-23	1	0	6	4–5	5–6	1–3(4)	2+2+3(4)	2	157–160	59–67
*A.hunanensis*	0.221 ~ 0.225	23	0.62 ~ 0.70	> 1	23-23-23	1	0	6	4–5	5–6	1–3(4)	2+2+4	2	163–165	69–72
*A.jinggangensis*	0.174 ~ 0.217	–	–	> 1	23-23-23	0	0	6	4–5	6	1–4	2(1)+2+3(4)	2	156–164	51–64
*A.juliani*	0.264 ~ 0.265	28	–	> 1	25-23-23	1	0	6	4–5	6	1–3(4)	2+2+4	2	163–169	81–91
*A.meiguensis*	0.142 ~ 0.238	17	–	–	(21–23)-(19–21)-(19–21)	1	1	6	4–5	6	1–3	2(3)+2(3)	1	146–173	39–60
*A.niger*	0.151 ~ 0.179	–	0.67	< 1	25-25-23	1	0	6	4–5	6	1–3(4)	2+2(3)	2	169–185	52–72
A.ningshanensis*	0.121 ~ 0.161	–	0.45 ~ 0.58	= 1	23-23-23(21)	1	0	6	4–5	5	1–2(3)	2+2(3)+3(4)	1–2	159–174	41–46
*A.panzhihuaensis*	0.246	28	0.67	–	23-23-19	1	1	6	4–5	6	1–3	2+2+3	1	160	73
*A.pingbianensis*	0.243	–	–	= 1	23-23-23	0	0	7	5–6	6	1–3	2+2+3	1	164	56
*A.rufescens*	0.191 ~ 0.221	23	0.90	> 1	23-(23–25)-23	1	0	6	4–5	5	1–3	2(1)+2+3(4)	1–2	148–156	58–69
*A.spinalis*	0.150 ~ 0.250	16–20	–	< 1	(23–25)-(23–25)-(23–25)	1	0	6	4–5	5–6	1–3	2+2(3)	1–2	138–175	48–67
*A.timi*	0.213	27	–	> 1	25-25-23	0	0	6	4–5	6	1–3	2+2+3	1	170	72
*A.tranganensis*	0.254(+)	29	–	> 1	25-23-23	1	0	6	4–5	6	1–3	2+2+3	2	171	73(+)
*A.vanhoensis*	0.264	32	–	> 1	25-23-23	0	0	6/7	4–5/5–6	6	1–4	2+3+3	2	176	84
*A.werneri*	0.250 ~ 0.300	–	–	= 1	?-(21–23)-?	1	0	6	4–5	6	–	2+3(4)	–	157–191	67–98
*A.yangdatongi*	0.261 ~ 0.262	26	0.57	> 1	23-23-23	1	0	6	4–5	5–6	1–3	2+2+2/3	2	155-161	76-82
*A.yunkaiensis*	0.185 ~ 0.203	20–24	0.49 ~ 0.64	= 1	23-23-23	1	0	6	4–5	6	1–3(4)	2+2+3(4)	2	150–162	49–56
*A.zugorum*	0.229	28	–	> 1	25-23-23	0	0	6	4–5	7	1–3	2+2+3	2	173	70

**Table 5.** Comparisons of main morphological characters of *Achalinusnanshanensis* sp. nov., *A.yangdatongi*, and *Achalinusdamingensis*.

	*A.nanshanensis* sp. nov.	*A.yangdatongi*		*A.damingensis*
Sex	**Males**	**Males**	**Females**	**Males**
SVL	300–392	241–293	292–424	322
TaL	99	85–104	73–93	105
TL	399–461	–	–	427
TaL/TL	**0.215–0.248**	**0.261–0.262**	0.180–0.200	**0.25**
HL	10.07–10.95	8.5–11.60	9.52–11.45	–
HW	5.96–7.25	–	–	–
MT	**18**	**26**	**24**	–
ED	1.09–1.11	–	–	–
SPL	6/6	6/6	6/6(rarely 5/5)	6/6
SPL-Eye	4^th^–5^th^	4^th^–5^th^	4^th^–5^th^	4^th^–5^th^
IFL	6	5/6	6	6/6
Chin	2	2	2	2
IFL-1^st^Chin	1^st^–3^rd^	1^st^–3^rd^	1^st^–3^rd^	1^st^–3^rd^
SPO	1	1	1	1
LorH / LorL	0.47–0.53	0.57	0.49	–
LSBI vs LSBP	>1	>1	>1	>1
TEM	**2+2(rarely 3)+4**	**2+2+2/3**	**2+2/3+2/3**	**2+2+3**
aTEM-Eye	2	2	2	2
DSR	23(25)-23(25)-23(25)	23-23-23	23-23-23	23-23-23
VEN	**147–158**	**155**	**170–171**	**165**
SC	64–77	76	59–64	74
Anal	1	1	1	1

The new species differs from *A.hunanensis* Ma, Shi, Xiang, Shu & Jiang, 2023 in having fewer ventral scales (147–158 vs 163–165), maxillary teeth (18 vs 23), and a bright-yellow collar around the neck.

The new species differs from *A.ningshanensis* Yang, Huang, Jiang, Burbrink, Gong, Yu, Zhang, Huang & Huang, 2022 in having more infralabials (6 vs 5), two pairs of chin shields (vs 3 pairs), LSBI significantly longer than LSBP (vs suture between internasals is similar in length when compared to the suture between prefrontals).

The new species differs from *A.ater* in having more temporals (2+2/3+4 vs 2+2+3), fewer ventral scales (147–158 vs 160–170), more SC (64–77 vs 47–70) and a bright-yellow collar around the neck.

The new species differs from *A.juliani* Ziegler, Nguyen, Pham, Nguyen, Pham, van Schingen, Nguyen & Le, 2019 in having fewer ventral scales (147–158 vs 173–179), fewer subcaudals (64–77 vs 77–91), and a bright-yellow collar around the neck.

The new species differs from *A.pingbianensis* Li, Yu, Wu, Liao, Tang, Liu & Guo, 2020 in having a separated loreal (vs loreal fused with prefrontal), more subcaudals (64–77 vs 56), LSBI significantly longer than LSBP (vs length of suture between internasals subequal to that between prefrontals), two anterior temporals in contact with the eye (vs only the upper anterior temporal in contact with the eye), fewer supralabials (6 vs 7), and a bright-yellow collar around the neck.

The new species differs from *A.timi* Ziegler, Nguyen, Pham, Nguyen, Pham, Van Schingen, Nguyen & Le, 2019 in having one loreal (vs no loreals), more infralabials (6 vs 5), temporals (2+2/3+4 vs 2+2+3), fewer ventral scales (147–158 vs 170), and fewer tooth (18 vs 27).

The new species differs from *A.vanhoensis* Ha, Ziegler, Sy, Le, Nguyen & Luu, 2022 in having fewer ventral scales (147–158 vs 176), fewer subcaudals (64–77 vs 84) and more temporals (2+2/3+4 vs 2+3+3).

The new species differs from *A.dabieshanensis* Zhang, Liu, Huang & Zhang, 2023, *A.huangjietangi* Huang, Peng & Huang, 2021, *A.niger* Maki, 1931 and *A.spinalis* Peters, 1869 by LSBI significantly longer than LSBP (vs suture between internasals). Furthermore, the new species differs from *A.dabieshanensis* in having more infralabials (6 vs 5). It differs from *A.huangjietangi* and *A.spinalis* in having more subcaudals in males (64–77 vs 59–67 and 64–77 vs 48–67, respectively). It differs from *A.niger* in having comparatively longer tail (0.215–0.248 vs 0.151–0.179).

The new species differs from *A.formosanus* Boulenger, 1908, *A.jinggangensis* Zong & Ma, 1983 and *A.zugorum* Miller, Davis, Luong, Do, Pham, Ziegler, Lee, De Queiroz, Reynolds & Nguyen, 2020 in having a separated loreal (vs no loreal). Furthermore, the new species differs from *A.formosanus* in having fewer dorsal scale rows (23–25)-(23–25)-(23–25) vs (29)27-27-25). It differs from *A.jinggangensis* in having more subcaudals (64–77 vs 51–64) and from *A.zugorum* in having fewer infralabials (6 vs7) and more temporals (2+2/3+4 vs 2+2+3).

The new species differs from *A.meiguensis* Hu & Zhao, 1966 in having more dorsal scale rows (23-23-23 vs (21–23)-(19–21)-(19–21), more subcaudals (64–77 vs 50–60), and having two pairs of chin shields (vs three pairs of chin shields).

The new species differs from *A.panzhihuaensis* Hou, Wang, Guo, Chen, Yuan & Che, 2021 in having more temporals (2+2/3+4 vs 2+3+3) and fewer ventral scales (147–158 vs 160).

The new species differs from *A.dehuaensis* Li, Wu, Xu, Zhu, Ren, Guo & Dong, 2021 in having more infralabials (6 vs 5) and fewer maxillary teeth (18 vs 31–33), and a bright-yellow collar around the neck.

The new species differs from *A.emilyae* Ziegler, Nguyen, Pham, Nguyen, Pham, van Schingen, Nguyen & Le, 2019 in having more infralabials (6 vs 5), temporals (2+2/3+4 vs 2+2+3), and a bright-yellow collar around the neck, and the new species differs from *A.emilyae* in the color of its dorsum (brownish-black vs pale yellowish brown).

The new species differs from *A.hainanus* Huang, 1975 in having more infralabials (6 vs 5), temporals (2+2/3+4 vs 1+2+3(4) and LSBI significantly longer than LSBP (vs suture between internasals similar size when compared to the suture between prefrontals).

The new species differs from *A.rufescens* Boulenger, 1888 in having more infralabials (6 vs 5) and two pairs of chin shields (vs three pairs of chin shields).

The new species differs from *A.tranganensis* Luu, Ziegler, Ha, Lo, Hoang, Ngo, Le, Tran & Nguyen, 2020 in having more temporals (2+2/3+4 vs 2+2+3) and fewer ventral scales (147–158 vs 171).

The new species differs from *A.werneri* Van Denburgh, 1912 in having a shorter tail (0.215–0.248 vs 0.250–0.300), fewer ventrals (147–158 vs 157–191) and LSBI significantly longer than LSBP (vs suture between internasals similar size when compared to the suture between prefrontals).

The new species differs from *A.yunkaiensis* Wang, Li & Wang, 2019 in having a comparatively longer tail in males (0.215–0.248 vs 0.185–0.203), more subcaudals (64–77 vs 49–56) and LSBI significantly longer than LSBP (vs suture between internasals similar size when compared to the suture between prefrontals).

## ﻿Discussion

With the discovery of *Achalinusnanshanensis* sp. nov., the number of *Achalinus* species now stands at 28, with 21 occurring in China: *A.ater* (Bourret, 1937), *A.dabieshanensis* (Zhang et al., 2023), *A.damingensis* (Yang et al., 2023), *A.dehuaensis* (Hou et al., 2021), *A.emilyae* (Ziegler et al., 2019), *A.formosanus* (Boulenger, 1908), *A.hainanus* (Huang, 1975), *A.huangjietangi* (Huang et al., 2021), *A.hunanensis* (Ma et al., 2023), *A.jinggangensis* (Zong & Ma, 1983), *A.meiguensis* (Hu & Zhao, 1966), *A.niger* (Maki, 1931), *A.ningshanensis* (Yang et al., 2022), *A.panzhihuaensis* (Hou et al., 2021), *A.pingbianensis* (Li et al., 2020), *A.rufescens* (Boulenger, 1888), *A.sheni* (Ma et al., 2023) *A.spinalis* (Peters, 1869), *A.nanshanensis* sp. nov. (this study), *A.yangdatongi* (Hou et al., 2021), and *A.yunkaiensis* (Wang et al., 2019). The genus *Achalinus* is known for its remarkable cryptic diversity, which has attracted extensive research. Nevertheless, some fundamental questions remain unanswered. Notably, a molecular comparison between populations of *A.ater* in Guangxi, China, and at its type locality at Tam Dao in northern Vietnam. Similarly, limited research has been made comparing *A.spinalis* found in China and at the type locality. This situation prompts us to reconsider the distribution of *A.ater* and *A.spinalis* in China.

Recent research has continued to underscore the remarkably high diversity within the genus *Achalinus*, which has lead to the discovery of an increasing number of species. However, several factors contribute to the difficulty in accurately identifying snakes of this genus based solely on morphology. *Achalinus* species display a conservative morphology; sexual dimorphism has been identified (particularly larger TaL/TL in males, more VEN in females, and more SC in males) (Ziegler et al. 2019; Hou et al. 2021; Huang et al. 2021; Li et al. 2021; Zhang et al. 2023); additionally, the possible existence of juvenile dimorphism has been suggested (Zhang et al. 2023). Furthermore, due to their secretive life history and morphological similarities, many cryptic species may well be hidden in plain sight within known widely distributed species (Ziegler et al. 2019; Li et al. 2020; Luu et al. 2020; Miller et al. 2020; Hou et al. 2021; Yang et al. 2022; Ma et al. 2023a; Yang et al. 2023). This poses a considerable challenge to future efforts aimed at comparing and identifying new species.

Molecular methods have played a pivotal role in the rapid discovery of *Achalinus* species (Yang et al. 2023). In addition, there are *A.yunkaiensis* and *A.sheni* distributed in the same region of *A.nanshanensis* sp. nov., which indicates that further study is necessary to conduct by using different geographic populations and molecular methods to revise their evolutionary history.

## Supplementary Material

XML Treatment for Achalinus nanshanensis

## ﻿Additional information

### Author ORCIDs

Hui Li https://orcid.org/0009-0009-6318-3223

Le-Qiang Zhu https://orcid.org/0009-0006-3820-529X

Bei Xiao https://orcid.org/0009-0006-0740-5360

Jie Huang https://orcid.org/0009-0005-2565-3109

Shao-Wu Wu https://orcid.org/0009-0001-1769-2088

Li-Xun Yang https://orcid.org/0009-0008-3664-5333

Zhi-Qiang Zhang https://orcid.org/0009-0004-8658-9625

Xiao-Yang Mo https://orcid.org/0009-0003-2352-8975

### Data availability

All of the data that support the findings of this study are available in the main text.
